# Systemic lupus erythematosus with refractory ulcerated livedoid vasculopathy: Successful treatment with intravenous immunoglobulin and warfarin

**DOI:** 10.1002/ccr3.1803

**Published:** 2018-09-12

**Authors:** Katsunobu Yoshioka, Chiharu Tateishi, Hiromi Kato, Ko‐Ron Chen

**Affiliations:** ^1^ Department of Internal Medicine Social Welfare Foundation Shitennoji Hospital Osaka Japan; ^2^ Department of Dermatology Osaka City University Graduate School of Medicine Osaka Japan; ^3^ Department of Nursing Social Welfare Foundation Shitennoji Hospital Osaka Japan; ^4^ Meguro Chen Dermatology Clinic Tokyo Japan

**Keywords:** intravenous immunoglobulin, livedoid vasculopathy, systemic lupus erythematosus, warfarin

## Abstract

We reported a patient with systemic lupus erythematosus complicated by livedoid vasculopathy (LV), who responded well to intravenous immunoglobulin and warfarin. Cutaneous lesions of LV resemble those of cutaneous vasculitis. LV should be included in the differential diagnosis of leg ulcerations even in the presence of autoimmune disorders.

## 1 INTRODUCTION

Livedoid vasculopathy (LV) is characterized by livedo reticularis and recurrent painful ulcerations.^1^ Histologically, LV shows thrombus formation and fibrin occlusion, involving dermal vessels, suggesting that the pathogenesis of LV is hypercoagulability.^2^ Therefore, anticoagulants are recommended. In addition, autoimmunity may be involved in the development of LV, because LV complicates various autoimmune diseases. Patients who have antiphospholipid antibodies with systemic lupus erythematosus (SLE) are particularly predisposed.^3^ Hence, immunosuppressive medications are occasionally used. However, these medications are often unsatisfactory.

Recently, intravenous immunoglobulin (IVIG) has shown to be effective in the treatment of LV.^4^, ^5^, ^6^, ^7^, ^8^, ^9^ However, a trial of IVIG for patients with SLE complicated by LV has not been done. We have successfully used IVIG and warfarin to treat a patient with SLE complicated by LV. This report provides review of our case and discusses the rationale for using IVIG in the treatment of LV.

## 2 CASE PRESENTATION

**Figure 1:**
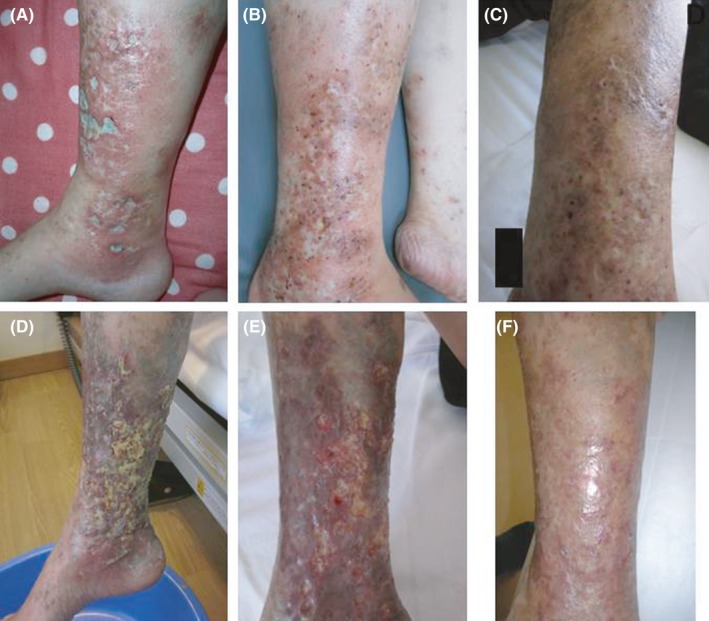
A, Right lower leg ulcerations at onset. B, Skin lesions at first deterioration, showing swelling of right leg with multiple small ulcerations before treatment. C, Complete healing with scars after treatment. D, Skin lesions at second exacerbation, showing swelling of right leg with multiple small ulcerations showing swelling of right leg with moth‐eaten appearance multiple ulcerations on admission. E, F, Serial changes of right leg ulcerations after intravenous immunoglobulin, arranged in temporal order

A 51‐year‐old woman was admitted to our hospital because of recurrent leg ulcerations. Eight years previously, she noticed purpura on both legs, which progressed to painful ulcerations. At that time, she was diagnosed with livedoid vasculitis complicated by cellulitis (Figure 1A). She noticed systemic joint pain and was referred to rheumatologist. Laboratory findings revealed positive for antinuclear antibody (×320), anti‐double‐strand DNA antibody (342 IU/mL), and anti‐cardiolipin antibody (ACA) (18 U/mL). She was diagnosed with SLE and treated with 15 mg of prednisolone together with topical therapies such as wound cleaning and topical ointments application. At this time, immunosuppressive agents were not used. Subsequently, leg ulcerations gradually improved and healed with scars in approximately 3 years.

She has remained asymptomatic under a maintenance dose of 10 mg of prednisolone However, leg ulcerations relapsed and she was referred to our hospital 2 years previously. Physical examination revealed swelling of right leg with multiple small ulcers, white scars, and purpura (Figure 1B). Deterioration of livedoid vasculitis complicated by SLE was suspected, and methylprednisolone pulse therapy (MPT: 1 g/d intravenously for 3 days) was introduced together with antiplatelet medications followed by 50 mg of prednisolone and 50 mg of azathioprine. Subsequently, she experienced immediate pain relief and leg ulcerations gradually improved and healed with scars in 2 months (Figure 1C).

**Figure 2:**
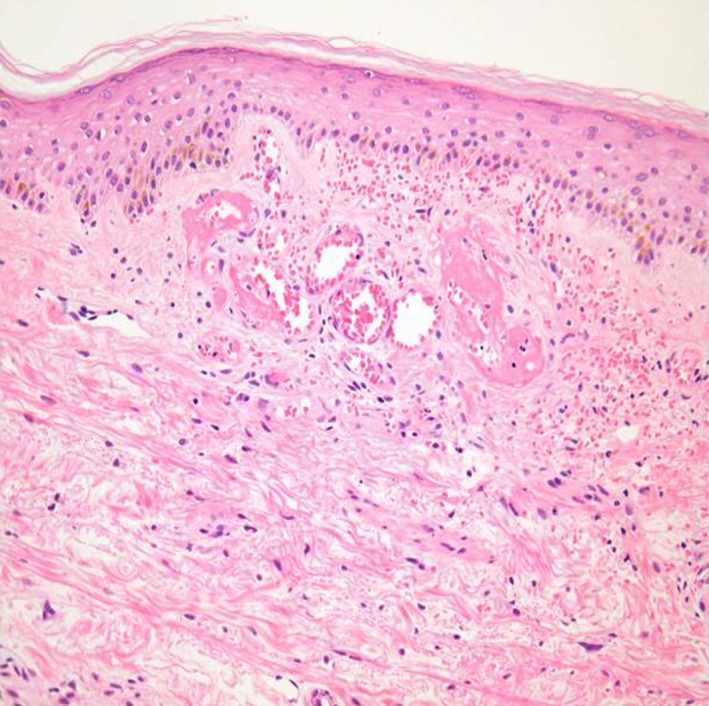
Light microscopic appearance of skin biopsy showing occlusion of superficial dermal small vessels due to fibrin thrombus. Infiltration of inflammatory cells around the dermal vessels is scarce (hematoxylin‐eosin staining, original magnification ×400)

Since healing of the ulcers, prednisolone was tapered and she has remained asymptomatic. However, 3 months previously, ulcerations relapsed on right leg. Physical examination revealed swelling of right leg with moth‐eaten appearance multiple ulcerations (Figure 1D). MPT had little effect this time. Skin rebiopsy revealed occlusion of superficial dermal small vessels due to fibrin thrombus. Infiltration of inflammatory cells around the dermal vessels was scarce (Figure 2). These findings were characteristic features of LV; thus, the diagnosis of LV was confirmed. We introduced IVIG (400 mg/kg of immunoglobulin for 5 days) together with warfarin to achieve international normalized ratio between 2 and 3. Subsequently, leg ulcerations gradually improved and healed with scars in 6 weeks (Figure 1E,F).

## 3 DISCUSSION

In addition to its anti‐inflammatory effects, it has been reported that IVIG has antithrombotic effects. The proposed mechanism of antithrombotic effects includes inhibition of thromboxane synthetase, thereby reduction in thromboxane A2 and decreasing the vasoconstriction,^10^ and inhibition of antiphospholipid antibodies. It is estimated that the combined anti‐inflammatory and antithrombotic effects of IVIG contribute to the treatment of LV in the present case.

The present case responded well to MPT without using warfarin when initial treatment was done. We consider the reason as follows: First, although skin biopsy could not reveal histological evidence of vasculitis, the skin lesion was actually vasculitis complicated by SLE; Second, antiphospholipid antibody syndrome may be related to the pathogenesis in the present case because ACA was positive. It is possible that MPT exerted as antithrombotic effects by inhibiting autoantibodies such as ACA, together with its anti‐inflammatory effects.

In summary, our results suggest that a trial of IVIG is warranted for patients with SLE complicated by refractory ulcerated LV.

## CONFLICT OF INTEREST

None declared.

## AUTHOR CONTRIBUTION

KY: reviewed medical records, interpreted data, and drafted the manuscript. CT: provided medical care and supervised the study, HK: provided medical care. KC: supervised the study.
